# Pilot Ex Vivo and In Vitro Evaluation of a Novel Foley Catheter with Antimicrobial Periurethral Irrigation for Prevention of Extraluminal Biofilm Colonization Leading to Catheter-Associated Urinary Tract Infections (CAUTIs)

**DOI:** 10.1155/2019/2869039

**Published:** 2019-12-23

**Authors:** Nylev Vargas-Cruz, Joel Rosenblatt, Ruth A Reitzel, Anne-Marie Chaftari, Ray Hachem, Issam Raad

**Affiliations:** Department of Infectious Diseases, Infection Control and Employee Health, University of Texas M.D. Anderson Cancer Center, Unit 1460, Houston, TX 77030, USA

## Abstract

CAUTI remains a serious healthcare issue for incontinent patients whose urine drainage is managed by catheters. A novel double-balloon Foley catheter was developed which was capable of irrigating the extraluminal catheter surfaces within the periurethral space between the urethral-bladder junction and meatus. The catheter has a retention cuff that is inflated to secure the catheter in the bladder and a novel irrigation cuff proximal to the urethral-bladder junction capable of providing periurethral irrigation from the urethral-bladder junction to the meatus. Uniform periurethral irrigation was demonstrated in an ex vivo porcine model by adding a dye to the antimicrobial urethral irrigation solution. An in vitro biofilm colonization model was adapted to study the ability of periurethral irrigation with a newly developed antimicrobial combination consisting of polygalacturonic acid + caprylic acid (PG + CAP) to prevent axial colonization of the extraluminal urethral indwelling catheter shaft by common uropathogens. The extraluminal surface of control catheters that were not irrigated formed biofilms along the entire axial urethral tract after 24 hours. Significant (*p* < 0.001) inhibition of colonization was seen against multidrug-resistant *Pseudomonas aeruginosa* (PA), carbapenem-resistant *Escherichia coli* (EC), and carbapenem-resistant *Klebsiella pneumoniae* (KB). For other common uropathogens including *Candida albicans* (CA), *Proteus mirabilis* (PR), and *Enterococcus faecalis* (EF), a first irrigation treatment completely inhibited colonization of half of the indwelling catheter closest to the bladder and a second treatment largely disinfected the remaining intraurethral portion of the catheter towards the meatus. The novel Foley catheter and PG + CAP antimicrobial irrigant prevented biofilm colonization in an in vitro CAUTI model and merits further testing in an in vivo CAUTI prevention model.

## 1. Introduction

The most common hospital-acquired infections are catheter-associated urinary tract infections (CAUTIs). They account for over 1 million cases annually including hospitals and nursing homes [1–3]. According to the Center for Disease Control National Healthcare Safety Network (NHSN), infections attributable to indwelling urinary catheters account for 75% of UTIs acquired in the hospital. Typical short- and long-term use of Foley catheters are common among patients with urinary retention due to bladder outlet obstruction, selected surgical procedures, prolonged incontinence, and urinary drainage for patients with prolonged immobilization [4]. CAUTIs often result in increased burdens to the patient as well as increased hospital stays and costs. More recently, prevention of CAUTI has become a major focus of many hospital infection control programs.

Pathogenesis of CAUTIs has been shown to be predominantly due to colonization of the indwelling Foley catheter sourced from organisms contaminating the urethral meatus [5, 6]. The subsequent development of biofilm by colonizing uropathogens is common in CAUTI since many express an array of adhesins and exopolysaccharides which promote attachment and biofilm formation on the catheter surface [7]. Both the internal and external surfaces of indwelling catheters are susceptible to biofilm colonization. There have been conflicting studies on the relative importance of the luminal pathway. One study with over 1000 patients inferred that the extraluminal pathway was more significant [5]; however, another study based on micrographic evidence concluded the intraluminal pathway was more important [8]. Both pathogenic routes can be important, so there remains a significant need for improved CAUTI prevention interventions for both surfaces. Here, we studied a novel method for disinfection and prevention of colonization of the extraluminal surface of Foley catheters. Blocking extraluminal colonization would isolate colonization to the intraluminal pathway, thereby enabling assessment of its relative importance for CAUTI prophylaxis. Optimal CAUTI prophylaxis might further be defined by future studies where the extraluminal methodology is combined with efficacious intraluminal interventions.

In previous studies, we have demonstrated in an in vitro biofilm colonization model [9] complete broad-spectrum biofilm eradication by the combination of 1% polygalacturonic (PG) acid and 0.4% caprylic acid (CAP). In order to disinfect the external surface of the Foley catheter, we created an irrigation solution treatment based on this combination (1% PG + 0.4% CAP). We also developed a novel double-cuff Foley catheter design that is capable of in situ irrigation of the external Foley catheter surface and periurethral space. The objective of this study was to assess an in vitro CAUTI model [6] that simulates the physiological environment of indwelling urinary catheters and prevention of biofilm formation by treating the external surfaces of Foley catheters with the 1% PG + 0.4% CAP irrigation solution.

## 2. Methods

### 2.1. Irrigation Solutions

Irrigation solutions were prepared, which contain 1% PG (<15% esterified; Sigma-Aldrich, St Louis, MO) and 0.4% CAP (Sigma-Aldrich, St Louis, MO). The pH of 1% PG solution was adjusted to pH 4.0–4.5, and 2-hydroxyethylcellulose (HEC) to a final concentration of 1.5% was added as a thickener. All irrigation solutions were added to a 3 ml syringe when utilized for in vitro testing.

### 2.2. Organisms Tested

A broad spectrum of highly virulent Gram-positive, Gram-negative, yeast, and chronic pathogenic clinical isolates from cancer patients in our hospital was selected for testing. These strains included multidrug-resistant (MDR) *Pseudomonas aeruginosa* (MDR-PA, MDA 118), carbapenem-resistant *Escherichia coli* (CRE-EC, MDA 123), carbapenem-resistant *Klebsiella pneumoniae* (CRE-KB, MDA125), *Candida albicans* (CA, MDA 117), *Proteus mirabilis* (PR, MDA 146), and *Enterococcus faecalis* (EF, MDA 183). All pathogens tested were clinical isolates selected from MD Anderson cancer patient cultures. Fresh organism cultures were grown from stored glycerol stocks on trypticase soy agar +5% sheep blood (for bacteria) or Sabouraud dextrose agar (for yeast) overnight at 37°C. For testing, pure culture was inoculated into Mueller Hinton broth (MHB) and diluted to 0.5 Mc Farland. Additional dilutions were made in MHB as necessary for testing.

### 2.3. Periurethral Irrigating Foley Catheter Design

**Figure 1:**
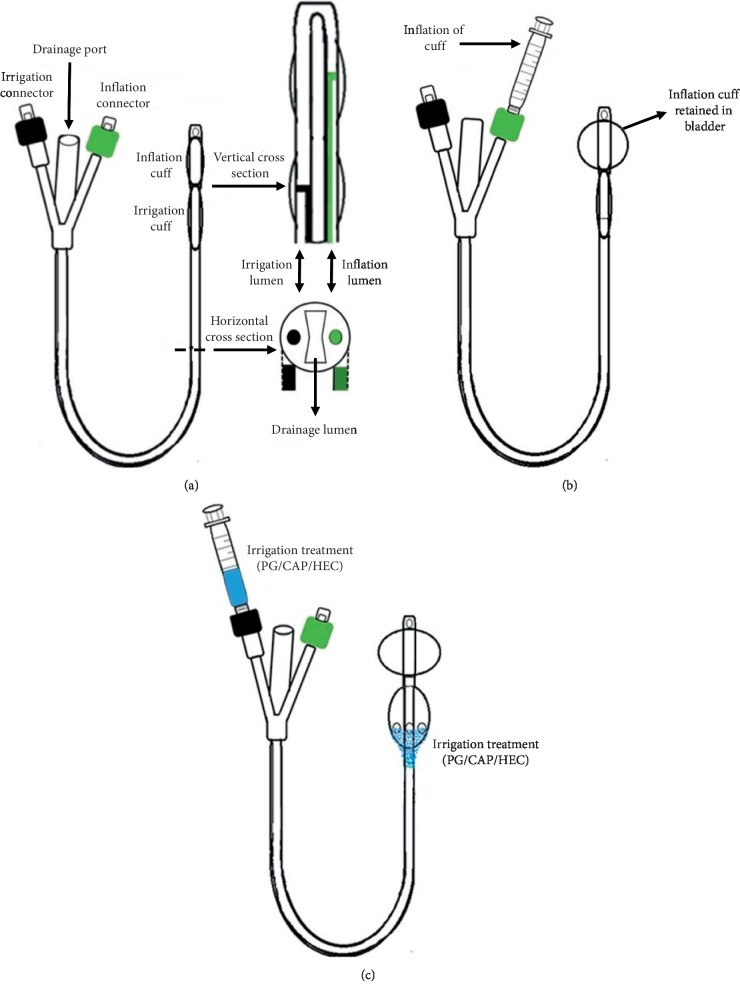
Double-cuff Foley catheter design. (a) Foley catheter design with vertical and horizontal cross sections showing lumens of double-cuff catheter. (b) Inflation of cuff retained within the bladder. (c) Addition of irrigation treatment in the second cuff.

A silicone Foley catheter was modified to enable retention in the bladder as well as to be able to apply irrigating solutions to the periurethral space. The design, illustrated in Figure 1, contained two cuffs: a distal cuff to retain the tip of the catheter in the bladder and a proximal irrigation cuff residing just below the urethral-bladder junction capable of disinfecting and lubricating or medicating the periurethral space contacting the external surface of the shaft of the catheter (Figure 1(a)). Each cuff has an independent inflation lumen in the wall of the catheter that axially traverses the catheter and terminates with a skived side-port opening through the external wall to where the cuff resides. The retention cuff has a longer lumen (shown in black on the left side of Figure 1(a)) than the irrigation cuff (shown in green on the right side of Figure 1(a)) Implantation of the Foley is performed with both cuffs deflated threading the tip and distal retention cuff into the bladder. The retention cuff is then inflated (Figure 1(b)) to retain the catheter tip in the bladder and to enable continuous bladder drainage through the eyelet at the catheter tip protruding slightly beyond the retention cuff. Like the retention cuff, the irrigation cuff is elastomeric; however, it has small slits at its base directed down the shaft of the catheter towards the meatus. Uniform irrigation of the periurethral space is accomplished by instillation of a viscous disinfecting solution (PG + CAP + HEC) through the irrigation port into the irrigation cuff (Figure 1(c)). The elastomeric irrigation cuff expands momentarily due to the pressure of the instilled liquid and then the elastomeric cuff pushes back on the instilled disinfecting solution pumping it through the irrigation slits at the proximal end of the cuff (Figure 1(c)) where it can irrigate the periurethral space contacting the external surface of the catheter shaft. Only small volumes of irrigation solution are instilled that are sufficient to coat the periurethral space but would not create significant irrigant discharge beyond the meatus. The HEC thickener was added to provide sufficient viscosity to uniformly coat the periurethral space with disinfecting solution during irrigation.

### 2.4. Ex Vivo Catheterization and Irrigation

The use of residual animal tissues was approved by MD Anderson Institutional Animal Care and Use Committee under protocol #00001666-RN00. Immediately postmortem following exsanguination, a 6-month-old female pig was placed sternally and catheterized with the double-cuff Foley catheter. The retention balloon was inflated to keep the catheter in place while slightly viscous irrigation solution to which a dye was added was infused through the irrigation cuff. The dye was added to visualize irrigation solution coverage of the periurethral space. After irrigation, a necropsy was conducted grossly removing the intact catheterized urinary tract including urethral meatus, urethra, and urinary bladder. The urinary tract was further dissected with a longitudinal opening along the length of the urinary tract to assess irrigant coverage.

### 2.5. In Vitro Foley Catheter Colonization Model (IVCM)

**Figure 2:**
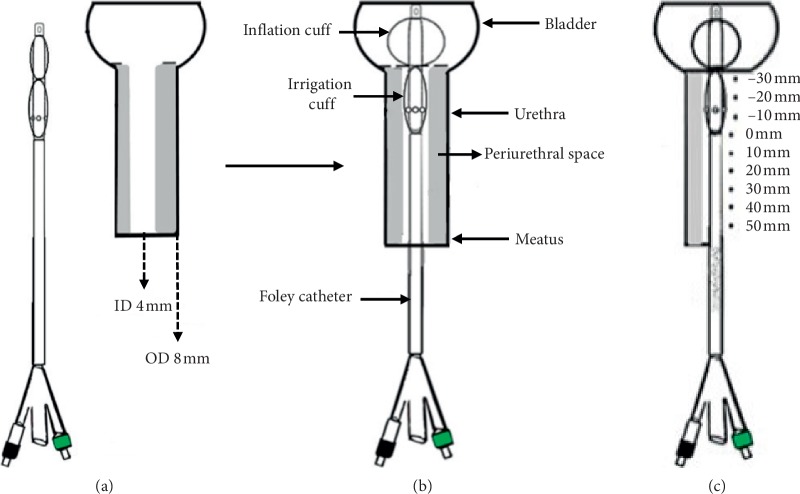
In vitro CAUTI model. (a) In vitro CAUTI model with Foley catheter out of the model. (b) In vitro CAUTI model with inserted Foley catheter (and retention cuff inflated postinsertion). (c) Length scaling of the Foley catheter inserted in the CAUTI model.

The Foley catheter with periurethral irrigation was evaluated in an established in vitro CAUTI prevention model [6]. Slight modification of the model was needed to simulate the physiological periurethral environment of the external surface of Foley catheters (Figure 2(a)). The model consisted of a soft 6 cm long silicone urethral tract through which the catheter could be threaded with cuffs deflated. The urethral tract consisted of a 4 mm annular opening at the center of a 25 mm diameter soft silicone cylinder (Figure 2(a)). The 4 mm annulus was slightly larger than the 12 French (3 mm outer diameter) Foley catheters that were utilized for testing. Figure 2(b) illustrates the positioning of the Foley catheter in the model where the retention cuff has been inflated to retain the catheter above the urethral-bladder junction. Figure 2(c) illustrates the length scaling of the model. Position “0” is taken as the proximal base of the irrigation cuff where the irrigation solution first contacts the periurethral surfaces. Distal to this point is denoted by negative lengths, where the urethral-bladder junction would correspond to position –10 mm and the drainage tip of the catheter residing in the bladder corresponds to length position –30 mm. Proximal to position “0” positive lengths denote axial positions in the urethra towards the meatus where positive 60 mm corresponds to the meatus.

The PG + CAP + HEC and control irrigation solutions were tested in the model to assess their effect on biofilm colonization of the external surface of the Foley catheter towards the bladder. Upon instillation of the irrigation solution to the irrigation cuff of the modified Foley catheter, the irrigation solution flowed through and filled the 60 mm periurethral space. Irrigation was halted when the first drops were seen emerging from the meatus, and the irrigation solution was allowed to slowly drain by gravity. Visible drainage was completed in about 15 minutes, but some residual irrigation solution coated the external surface of the catheter shaft.

### 2.6. Biofilm Colonization Experiments

#### 2.6.1. Biofilm Formation

**Figure 3:**
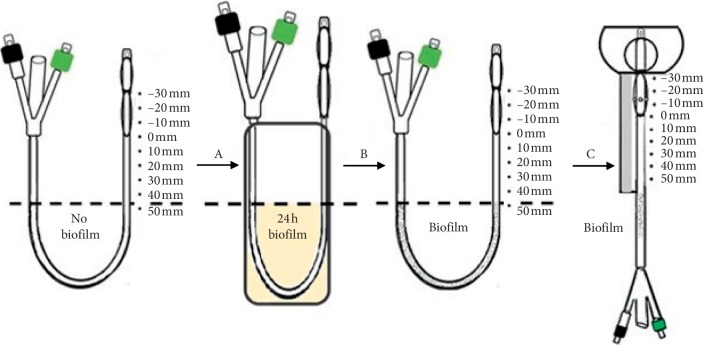
Biofilm formation—Step A: “U” portion of double-cuff Foley catheter shaft is suspended in Mueller Hinton broth containing uropathogen inoculum and incubated at 37°C for 24 h. Step B: biofilm is formed on the submerged portion of double-cuff Foley catheter shaft. Step C: Foley catheter with shaft partially colonized with biofilm is inserted in the in vitro CAUTI model. The biofilm extends up to the meatal opening of the model; the intraurethral dwelling portion of the shaft (90 mm long shaft section with positions denoted +50 mm to –30 mm in the figure) is not colonized at the time of insertion. The position denoted +50 mm corresponds to the meatus and the 0 mm position corresponds to the entry position of periurethral irrigation solution near the urethral-ladder junction. The periurethral irrigation occurs downwards over a 50 mm section of catheter shaft from the 0 mm position to the +50 mm position.

**Figure 4:**
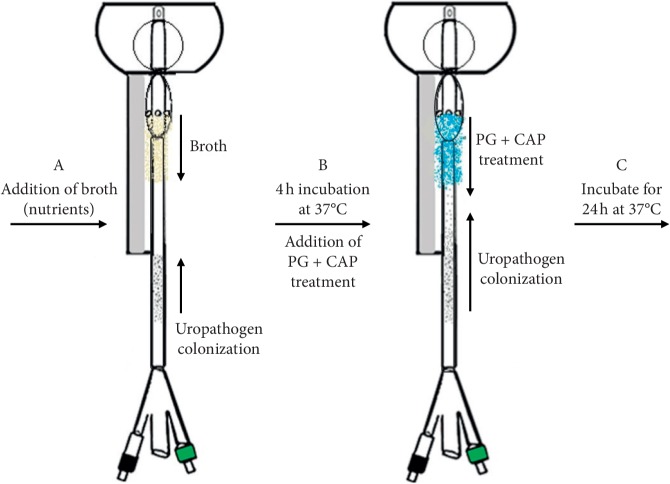
Illustration of periurethral irrigation in vitro experiment—Step A: the Foley catheter with 24 h biofilm preformed on catheter shaft proximal to the meatus is inserted model for evaluation. Inflation of retention cuff in bladder retains the shaft in the urethra. Step B: irrigation of the periurethral space with diluted Mueller Hinton broth wets the catheter shaft and incubation for 4 hours provides nutrients for biofilm to colonize transurethrally up to the bladder. Step C: controls were incubated for an additional 24 h at 37°C. Treatment catheters were first irrigated with a small volume of PG + CAP irrigation solution and then incubated for 24 h at 37°C. In some experiments, a second PG + CAP irrigation treatment was performed 24 hours after the first and incubated a further 24 hours at 37°C prior to quantifying viable colonies on the transurethral portion of the catheter shaft.

Biofilms of test uropathogens were formed on the catheters distal to the “urethral meatus” as described in Figure 3 prior to initiation of an irrigation experiment. The catheters were suspended above a flask containing a test uropathogen in MHB. The catheters were suspended such that a “U” was immersed in the inoculum. The distal end 90 mm containing the tip and cuffs was not immersed (Figure 3, Step A), and the proximal end containing the drainage bag connector and inflation ports was not immersed as well. The submerged “U” section of the Foley catheter was allowed to form biofilm overnight at 37°C (Figure 3, Step B). After 24 h biofilm formation, the distal end of the catheter was inserted in the CAUTI model and the retention cuff inflated (Figure 3, Step C). 1 ml of Mueller Hinton broth with 2-hydroxyethylcellulose (HEC) was then irrigated and incubated for 4 h at 37°C to wet and provide nutrients for the biofilm to be able to colonize towards the bladder (Figure 4, Steps A and B). Subsequently, 2 ml of experimental irrigation solution (1%PG + 0.4%CAP + 1.5%HEC) was infused through the irrigation cuff and allowed to drain through the periurethral space along the external surface of the catheter and incubated for an additional 24 hours (Figure 4, Step C) following which axial biofilm concentrations were assessed. In some experiments, a second irrigation was performed following the first irrigation and 24-hour incubation. These catheters were then allowed to incubate an additional 24 hours following which axial biofilm concentrations were assessed.

#### 2.6.2. Quantitative Assessment of Axial Biofilm Density

**Figure 5:**
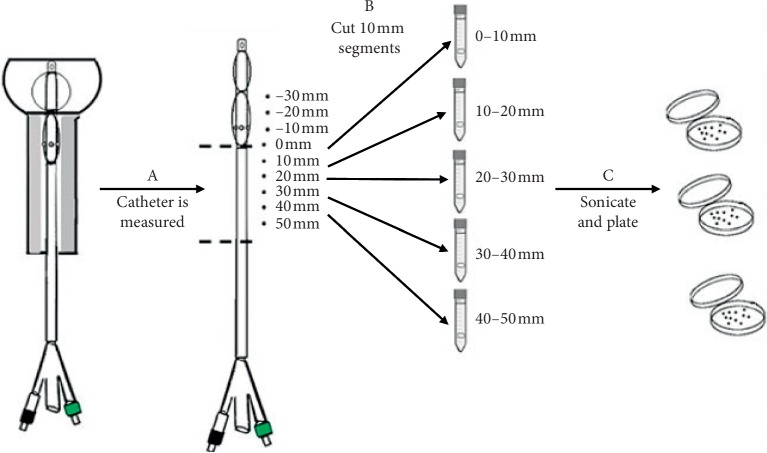
Enumeration of viable colonies method—Step A: catheter is taken out of the in vitro model and the shaft was cut into 10 mm segments from the base of the irrigation cuff to the meatus (Step B). The 10 mm segments are added to 5 ml of saline water. Step C: segments are sonicated for 15 minutes, serially diluted, and plated in corresponding media plate. The plates were incubated for 24–48 hours, and then viable colonies were enumerated.

The Foley catheter was then cut into 10 mm segments (6 samples) from the meatus towards the urethral-bladder junction. Each segment was placed in 5 ml of 0.9% of saline and sonicated for 15 minutes. Samples were serially diluted and plated onto trypticase soy agar +5% sheep blood (bacteria), Sabouraud dextrose agar (yeast), or eosin methylene blue agar (*Proteus*). Plates were incubated inverted for 24 hours at 37°C and counted for viable colonies recovered from the sonicated segments (Figure 5). Negative controls were sterile Foley catheters that were not challenged.

#### 2.6.3. Statistics

To determine whether there was a significant difference in colonization of the segment closest to the bladder (40–50 mm from urethral meatus) between irrigation solutions, the Kruskal–Wallis test was used. Pairwise comparisons were assessed using the Mann–Whitney *U* test to compare the performance of 1 or 2 flush PG + CAP irrigation solutions to control. All tests were two-sided with an alpha level of 0.05. A *p* < 0.05 was used to determine significance.

## 3. Results

**Figure 6:**
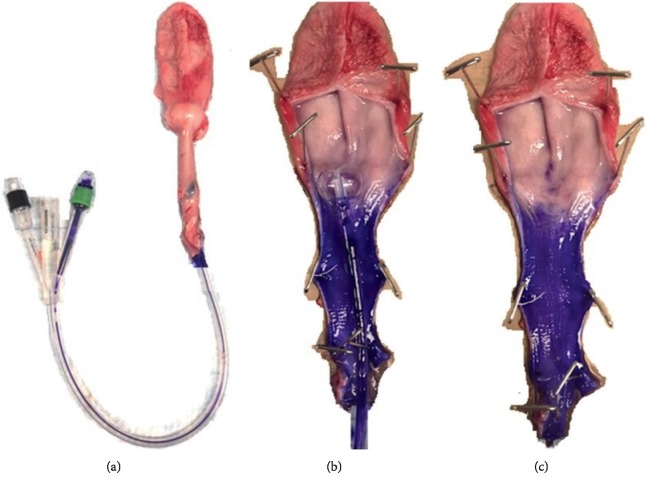
Photographs following the ex vivo irrigation experiment. (a) The excised intact urinary tract with inflated retention cuff visible at the base of the bladder and the catheter shaft distending the urethra and exiting at the meatus. A drop of violet dye is present proximal to the meatus showing the exit site of the irrigation solution. (b) The transversely opened urethra and bladder with catheter still present. The violet dye shows the portion of the urethra that was contacted by the irrigation solution. (c) The transversely opened urethra and bladder with catheter removed to provide unobstructed view of the uniform coverage of the periurethral space by the irrigation solution.

The double-cuff Foley catheter was placed in the urinary tract of a porcine cadaver and irrigated with an irrigation solution (PG + CAP + HEC) to which a violet dye was added to aid in visualizing the irrigant (Figure 6). Figure 6 shows the urinary tract that was excised from the pig (Figure 6(a)), how the irrigation cuff is retained in the bladder (Figure 6(b)), and the portions of the urethra that were treated by irrigation solution are visible with the presence of violet dye (Figure 6(c)). The entire urethra and periurethral space proximal to the irrigation cuff was uniformly treated and coated with dye from the irrigation solution.

**Figure 7:**
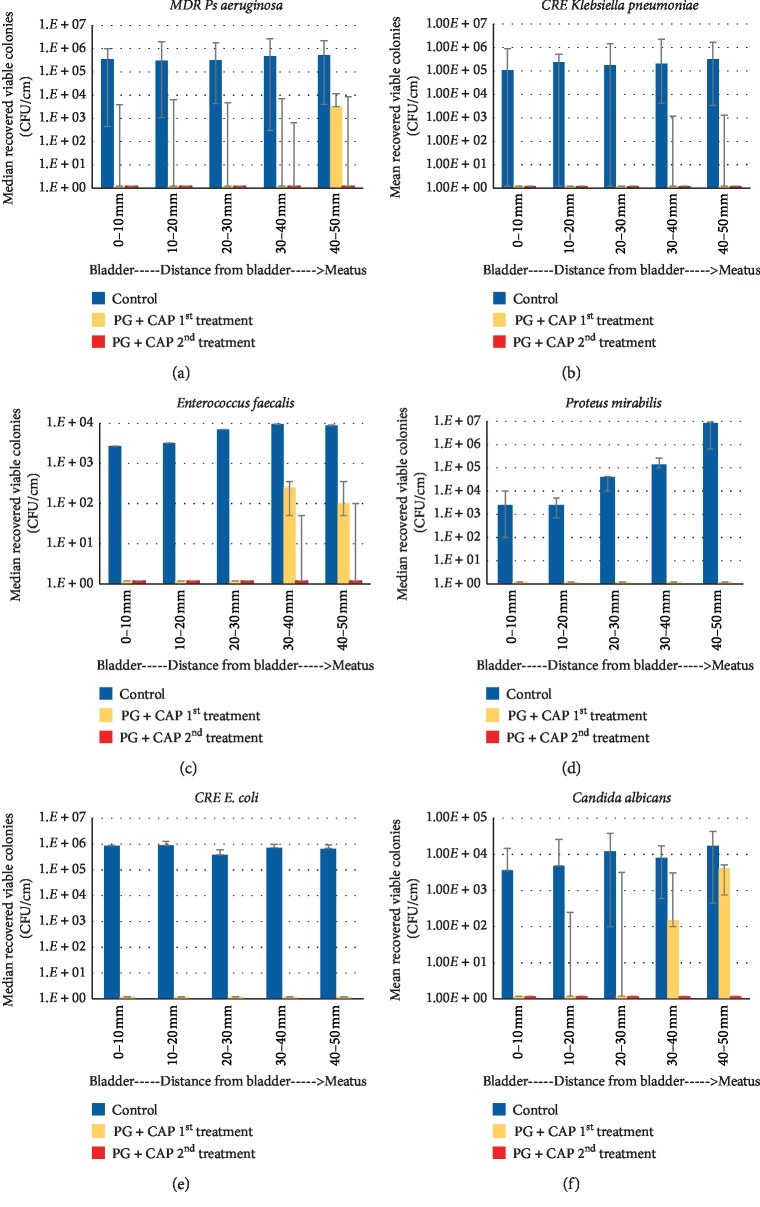
Efficacy of PG + CAP irrigation treatment in CAUTI model. Median viable colonies recovered (and ranges) from catheter shafts (3 replicates) sectioned at different axial positions (50 mm corresponding to the meatus and 0 mm corresponding to near the urethral-bladder junction) following in vitro biofilm colonization experiments. (a) MDR-PA, (b) CRE-KB, (c) EF, (d) PR, (e) CRE-EC, and (f) CA. Control catheters, first PG + CAP irrigation treatment, and second PG + CAP irrigation treatment are denoted by different colors.

Results for median and ranges (Figure 7) of axial biofilm colonization present for the different uropathogens tested. Almost all of the control catheters were able to be colonized along the external surfaces of the catheters up to the base of the irrigation cuff (0–10 mm) adjacent to the bladder. For *E. coli* and *Pseudomonas*, the biofilm concentrations of the positive controls were axially uniform from the meatus (40–50 mm) to the bladder (0–10 mm). These organisms were rapid colonizers. For other organisms (CA, PA, KB, and EF), there was a slightly lower axial biofilm concentration at the bladder (0–10 mm) than at the meatus (40–50 mm) within 1–2 log_10_s. For PR, there was a more pronounced axial gradient where there was greater than a 3-log_10_ difference between the meatus (densest biofilm) and the bladder. A single irrigation treatment of PG + CAP irrigant was able to fully prevent colonization of the external surface of the catheter within the urethral tract for EC and PR. Colonization of 0 mm to 30 mm range closest to the bladder was prevented for the other organisms on the first irrigation treatment. The second irrigation treatment was able to extend the uncolonized zone to the full 50 mm of the urethral tract for all other organisms. One irrigation treatment significantly prevented colonization by the Gram-negative organisms *Pseudomonas*, *E. coli*, and *Klebsiella* compared with control Foley catheters (*p* < 0.001).

## 4. Discussion

Use of double-cuff Foley catheters has been previously reported where the second cuff was placed in various positions for a variety of purposes. These include secondary cuffs at the drainage tip to cushion the bladder wall [10, 11], catheters with secondary cuffs for surgical management and manipulation [12, 13], and catheters with secondary cuffs distal to the meatus for diagnostic purposes [14, 15]. A double-cuff catheter capable of irrigating the external catheter shaft surface, and the periurethral space was reported here and was able to be inserted using routine Foley catheter insertion practices. The retention cuff was able to be inflated and retain the catheter tip in the bladder for continuous urine drainage. By adding a dye to aid in visualization, a PG + CAP antimicrobial irrigation solution slightly thickened with 2-hydroxyethylcellulose (HEC) was able to be delivered through the irrigation cuff using a standard syringe and with several slits evenly spaced around the base of the irrigation cuff. The catheter design was further able to directionally deliver the irrigation solution in a manner that uniformly coated the periurethral space from the base of the irrigation cuff near the urethral-bladder junction to the meatus. In the experiments, a slight excess of irrigation fluid was employed to ensure the complete length of the periurethral tract along the catheter shaft was treated. In practice, use of an excess volume of irrigant might create some exudate beyond the meatus that would need to be absorbed or cleaned. This can be reduced by irrigating a smaller volume or by immediately following the irrigation with routine meatal care and cleansing where any discharge below the meatus can be absorbed and removed with a meatal cloth or gauze.

In order for external surface sourced CAUTI to occur, pathogenic microbes must colonize and migrate along the catheter shaft to reach the bladder [5, 16]. This typically takes a few days in a newly catheterized patient. Preventing microbes from accessing the bladder by maintaining a decolonized zone between the urethral-bladder junction and meatus would consequently prevent the bladder from getting infected via this pathway. An in vitro CAUTI model was modified here to test the effectiveness of the mechanism of disinfection by external surface irrigation for preventing colonization up to and beyond the urethral-bladder junction along the catheter shaft.

There are many parallels and some differences between the in vitro model and the physiologic situation. Following catheter insertion, the periurethral space is relatively quiescent. There can be some slight leakage of urine and other fluids into the periurethral space towards the meatus, but there is no significant convective flow. Because of the distensibility of the urethra and tendency to collapse against the catheter shaft, the periurethral space remains moist without any physical barriers preventing the migration of microbes towards the bladder. In the model, the periurethral space is maintained moist and there is a small gap between the simulated soft silicone urethra and catheter in place of distensible contact. The physiologic system is not necessarily straight while the model is, the physiologic length between meatus and bladder can be longer than 60 mm, and then a wetting of microbial nutrients is not present to encourage colonization towards to bladder. This might be the primary reason for the difference in time to fully colonize the catheter shaft; in the case of the model, it took one day whereas in the physiologic system it typically takes at least an extra day [17].

The PG + CAP antimicrobial combination was previously shown to be effective in eradicating biofilms of a wide range of organisms [9]. We also demonstrated that it had very low cytotoxicity towards human fibroblasts. Caprylic acid is a naturally occurring fatty acid present in significant quantities in breast milk, coconut oil, and other natural fluids. It has been designated generally regarded as safe by regulatory agencies and has been safely used in intravenous parenteral nutrition formulations, as a dietary supplement, and topically for skin, hair, and wound care [9, 18]. Polygalacturonic acid is naturally derived from pectin and typically isolated from citrus rind of apple pomace [18]. Polygalacturonic acid is also generally regarded as safe and has been used medically in hydrocolloid wound care dressings and as an emulsifying pharmaceutical excipient in a range of pharmaceutical formulations [9]. Here, we showed that it could be placed in a thickened fluid that could be delivered through the external surface irrigating Foley catheter where it could flow in a countercurrent direction to the direction of colonization. PG + CAP in the slightly viscous irrigation vehicle was able to prevent colonization up the catheter shaft in the model to the level of the urethral-bladder junction. Since antibiotic resistance has been problematic with biofilm-associated CAUTI [19], the PG + CAP combination presents a nonantibiotic treatment that would not contribute to induction of antibiotic-resistant pathogenic microbes.

For a few organisms (*C. albicans, MDR Pseudomonas aeruginosa*, *and Enterococcus faecalis*), a second treatment was needed to fully decontaminate to the level of the meatus. For CRE *Klebsiella pneumoniae*, the entry 10 mm beyond the meatus remained colonized following both treatments; there was however about a 3-log_10_ reduction in the density of bacteria compared to the control in this entry segment and the remaining 50 mm between the urethral-bladder junction and entry segment of the catheter was sterile. This suggests that daily disinfection treatments with PG + CAP irrigation would be needed and potentially effective in disinfecting and maintaining a sterile zone along the catheter shaft between the meatus and bladder. This intervention furthermore would have the potential to prevent external surface sourced CAUTI.

Other interventions have been proposed for minimizing both extraluminal and intraluminal colonization pathways with conflicting reports and guidance for effectiveness in preventing CAUTI. The effectiveness of antimicrobial irrigation of the bladder followed by drainage had been contested in CDC guidelines [20] but more recently reported effective elsewhere [21, 22]. Effectiveness of antimicrobial catheter coatings has similarly been reported to be inconclusive [23–25]. CDC guidelines contest the effectiveness of meatal disinfection, but in recent study reports it was effective [26]. Use of antimicrobial lubricating gels for catheter insertion has been suggested to be ineffective [27, 28], but in some cases, it can have benefits [29]. Locking Foley catheters with antimicrobial solutions has been proposed as an effective method for disinfecting the intraluminal surfaces [30, 31] but has yet to be assessed clinically. Some of the apparently conflicting clinical outcomes could be a result of the inability to isolate the pathogenic pathway most affected by the intervention being assessed. Combining specific intraluminal disinfection interventions with the extraluminal disinfection method presented in the study would potentially enable isolation of colonization to the intraluminal pathway, thereby enabling independent identification of the most effective intraluminal interventions. In conclusion, we presented a novel double-cuff Foley catheter that was capable of being retained in the bladder to accomplish urinary drainage as well as being capable of irrigating the periurethral space and external surface of the intraurethral catheter shaft. The performance was demonstrated in an ex vivo model. As a prelude for testing for CAUTI prevention, we adapted an in vitro model for external surface microbial colonization of intraurethral Foley catheter shafts. Irrigation treatments with PG + CAP were capable of preventing in vitro colonization through the urethra with daily irrigation treatments. This system also presents the potential for preventing or treating other urethral tissue morbidities in chronically catheterized patients by the addition of additional agents to the irrigant solution. The catheter and PG + CAP irrigation system warrants further study in in vivo CAUTI models.

## Data Availability

All data related to this submission are available by request to authors.
